# Comparison and Evaluation of Four Species of Macro-Algaes as Dietary Ingredients in *Litopenaeus vannamei* Under Normal Rearing and WSSV Challenge Conditions: Effect on Growth, Immune Response, and Intestinal Microbiota

**DOI:** 10.3389/fphys.2018.01880

**Published:** 2019-01-09

**Authors:** Jin Niu, Jia-Jun Xie, Tian-Yu Guo, Hao-Hang Fang, Yan-Mei Zhang, Shi-Yu Liao, Shi-Wei Xie, Yong-Jian Liu, Li-Xia Tian

**Affiliations:** State Key Laboratory of Biocontrol, Institute of Aquatic Economic Animal and Guangdong Province Key Laboratory for Aquatic Economic Animals, School of Life Sciences, Sun Yat-sen University, Guangzhou, China

**Keywords:** *Litopenaeus vannamei*, macro-algaes, growth, immune response, intestinal microbiota, WSSV challenge test

## Abstract

The study was conducted to compare and evaluate effects of four different macro-algaes on growth, immune response, and intestinal microbiota of *Litopenaeus vannamei*. In the rearing trial 1, shrimp were fed five diets containing four sources of macro-algaes for 8 weeks, named D1 (without macro-algae), D2 (*Porphyra haitanensis*), D3 (*Undaria pinnatifida*), D4 (*Saccharina japonica*), and D5 (*Gracilaria lemaneiformis*), respectively. Growth performance of shrimp in D5 diet was significantly higher than that of shrimp fed the control and D4 diet (*P* < 0.05); however, there is no significant difference among D2, D3, and D5 diets (*P* > 0.05). Apparent digestibility coefficients of dry matter from the D2, D3, and D5 diets were significantly higher than that from the control and D4 diets (*P* < 0.05). Supplementary macro-algaes enhanced hepatopancreas immunity through positively increasing total antioxidant status (TAS) and prophenoloxidase activity (ProPO), as well as up-regulating the hepatopancreas RNA expression of ProPO and IκBα and down-regulating the expression of transforming growth factor β. Furthermore, dietary macro-algaes modified intestinal microbiota of *L. vannamei*, boosting the relative abundance of beneficial bacterial such as *Bacteroidetes, Firmicutes*, and *Bacillaceae*, and decreasing those detrimental bacterial such as *Gammaproteobacteria* and *Vibrionaceae*. In the white spot syndrome virus (WSSV) challenge trial, shrimps were injected for 6-day after the rearing trial. On the fourth day, shrimp death started to occur, and the mortality in D2, D3, and D5 diets was significantly lower than that in control and SJ diets during 4–6 challenged days (*P* < 0.05). Dietary macro-algaes ameliorated hepatopancreas damage in *L. vannamei* by increasing TAS and ProPO activities and decreasing SOD activity, inhibiting the lipid peroxidation (malondialdehyde), as well as regulating the immune-related genes expression. Taken together, dietary macro-algaes availably relieved enterohepatic oxidative damage by improving antioxidant ability and immunity and regulated intestinal microbiota in *L. vannamei*. These results indicated that *G. lemaneiformis* is the most suitable macro-algae and then followed by *U. pinnatifida* and *P. haitanensis* as the feed ingredient for *L. vannamei*.

## Introduction

Macro-algae were used to replace animal ingredients in terrestrial animal feeds (Younis et al., 2018), as a source of pigments (Chakdar and Pabbi, 2017), to enhance antioxidant and anti-inflammatory activities (Shogo et al., 2015), to improve gut function (Michiels et al., 2012), etc. Seaweeds are now gaining increasing attention due to the abundant bioactive compounds and nutrition (Niu et al., 2015; Cabrita et al., 2016), which were used in rainbow trout, *Oncorhynchus mykiss* (Güroy et al., 2011; Sheikhzadeh et al., 2012), sea bass, *Dicentrarchus labrax* (Valente et al., 2006), guppy, *Poecilia reticulata* (Nath et al., 2012), *Penaeus monodon* (Niu et al., 2015), and *Litopenaeus vannamei* (Yu et al., 2016; Boonsri et al., 2017; Schleder et al., 2017) as a dietary supplement.

To some extent, feeding algaes has led to enhanced performance, including improved pellet quality, feed efficiency, and animal product quality.

Researches have investigated seaweeds about the nutritional and nutraceutical effects and the function as binder effect with possible usefulness (Valente et al., 2006; Niu et al., 2015; Yu et al., 2016; Schleder et al., 2017). However, the optimum inclusion level may differ from species of algaes or consumers. Valente et al. (2006) showed that worse growth performance occurred in sea bass with diet containing over 10% of *Gracilaria bursa pastoris*. Niu et al. (2015) revealed that the optimal inclusion of *Undaria pinnatifida* (UP) in diet for *P. monodon* should be 2.17–2.87%. Yu et al. (2016) indicated that the *L. vannamei* fed with diets supplemented with *Gracilaria lemaneiformis* (GL) at 2–3% had significantly higher growth performance than shrimp fed diets containing 0–1% or 4–5% GL. Schleder et al. (2017) found that *L. vannamei* fed diets supplemented with lower *Sargassum filipendula* showed higher cumulative survival compared to those fed the control and higher inclusion diets after thermal shock.

Production of macro-algaes has been increasing in the last few decades but there is limitation to a few species which are able to cultivate on a commercial scale (Nagler et al., 2003). However, China is rich in algae resource with more than 100 kinds of algaes considered to have economic values. Several species of macro-algae have already been used for the potential ingredients in the diet of shrimp, such as *Cryptonemia crenulata* (Silva and Barbosa, 2009), *Gracilaria cervicornis* (Marinho-Soriano et al., 2007), *Gracilaria parvispora* and *Ulva lactuca* (Rodríguez-González et al., 2014; Pallaoro et al., 2016), *Hypnea cervicornis* and *Ulva clathrata* (Peña-Rodríguez et al., 2011), UP (Niu et al., 2015; Schleder et al., 2017). However, to our knowledge, there were no researches about the economic seaweeds, *Porphyra haitanensis* (PH), *Saccharina japonica* (SJ), and GL as ingredients in shrimp feeds, let alone compare the additional effect of them to the shrimp diet simultaneously. The utilization of different algae species in the feeding of shrimp should be compared.

The pacific white shrimp is considered to be one of the most important aquaculture species all over the world (Food and Agriculture Organization [FAO], 2010). There are many studies about the effects of individual macro-algae on growth performance and immune response in *L. vannamei* (Yu et al., 2016; Niu et al., 2018; Schleder et al., 2018). However, the comparison among different kinds of macro-algae on pacific white shrimp was not found. Therefore, the present study was to evaluate the four marine seaweeds, *Porphyra haitanensis, Undaria pinnatifida, Saccharina japonica*, and *Gracilaria lemaneiformis* as dietary ingredients on growth performance, immune response, intestinal microbiota, and the resistance to WSSV injection.

## Materials and Methods

### Experimental Diets and Diets Preparation

All the four macroalgaes samples were supplied by National Algae Project Technology Research Center of China. The four macro-algaes samples were dried and finely ground. Nutrient compositions of the four macro-algaes meals are shown in Table 1. The formulation were shown in Table 2 with four different species of macro-algaes supplemented. D1 as the control diet did not add any macro-algae. D2–D5 diets were added PH, UP, SJ, and GL, respectively. Diets were prepared according to Niu et al. (2015).

**Table 1:** Nutrient composition of the experimental algae meals.

	*Porphyra*	*Undaria*	*Saccharina*	*Gracilaria*
	*haitanensis*	*pinnatifida*	*japonica*	*lemaneiformis*
Protein (%)	33.60	11.60	8.00	13.69
Lipid (%)	0.40	3.74	0.60	0.44
Ash (%)	6.62	18.93	23.99	6.20
Carotenoids (μg/g)	5.49	9.10	1.58	98.00

*Value are based on dry weight.*

**Table 2:** Composition and nutrient levels of five experimental diets (%DM basis).

Items	D1	D2	D3	D4	D5
Ingredients					
Fish meal	25	25	25	25	25
Soybean meal	22	22	22	22	22
Peanut meal	12	11	11	11	11
Wheat flour	23.39	22.39	22.39	22.39	22.39
Beer yeast	5	5	5	5	5
Krill meal	5	5	5	5	5
Soya lecithin	1	1	1	1	1
Fish oil	1	1	1	1	1
Soybean oil	1	1	1	1	1
Ca(H_2_PO_4_)_2_-H_2_O	1	1	1	1	1
Vitamin premix^1^	1	1	1	1	1
Mineral premix^2^	1	1	1	1	1
Ascorbic phosphate ester	0.1	0.1	0.1	0.1	0.1
Choline chloride (50%)	0.5	0.5	0.5	0.5	0.5
*Porphyra haitanensis*	0	2	0	0	0
*Undaria pinnatifida*	0	0	2	0	0
*Saccharina japonica*	0	0	0	2	0
*Gracilaria lemaneiformis*	0	0	0	0	2
Sodium alginate	1	1	1	1	1
Yi_2_O_3_	0.01	0.01	0.01	0.01	0.01
Total	100	100	100	100	100
Nutrient levels^3^					
Moisture	9.03 ± 0.01	9.16 ± 0.03	9.09 ± 0.07	9.12 ± 0.17	9.20 ± 0.04
Crude protein	40.19 ± 0.15	40.25 ± 0.17	39.89 ± 0.06	40.27 ± 0.23	39.99 ± 0.06
Crude lipid	6.44 ± 0.12	6.54 ± 0.05	6.44 ± 0.05	6.45 ± 0.04	6.45 ± 0.06
Ash	11.32 ± 0.03	11.65 ± 0.01	11.73 ± 0.03	11.88 ± 0.01	11.52 ± 0.04

*^*1*^Vitamin premix provides the following per kg of diet: VD_*3*_ 0.6 M.I.U., VB_*1*_ 3.6 g, VB_*2*_ 7.2 g, VB_*6*_ 6.6 g, VB_*12*_ 0.02 g, VE 16.5 g, VK_*3*_ 2.4 g, VB_*3*_ 14.4 g, VB_*5*_ 4 g, biotin 0.02 g, folic acid 1.2 g, inositol 30 g, Vc 100 g, and cellulose was used as a carrier (according to Niu et al., 2015).*

*^*2*^Mineral premix provides the following per kg of diet: P 120 g, Ca 120 g, Mg 15 g, Fe 1.5 g, Zn 4.2 g, Cu 2.1 g, K 75 g, Co 0.11 g, Mn 1.6 g, Se 0.01 g, Mo 0.005 g, Al 0.025 g, I 0.4 g, and cellulose was used as a carrier (according to Niu et al., 2015).*

*^*3*^Measured values.*

### Shrimp and Experimental Set Up-Trial 1

The feeding trial was conducted at Sanya, Hainan Province. Before the trial, *L. vannamei* juveniles were acclimated to a control diet for 2 weeks. After the acclimatization period, 600 shrimps were starved for 24 h, weighed, and randomly distributed to 15 fiberglass tanks with similar size (IBW 0.65 ± 0.01 g).

The feeding strategy was the same as Niu et al. (2018). The feeding period lasted for 56 days. After the rearing trial, feces were collected for apparent digestibility coefficiencts analysis according to the method described by Niu et al. (2012).

### Macro-Algaes, Diet, and Shrimp Samples Composition Analysis

All samples were dried and grounded. The measurement of macro-algaes carotenoids was conducted according to the method of Schierle and Hardi (1994). Moisture, crude protein, crude lipid, and crude ash of the macro-algaes, diets, fecal, and shrimp were analyzed by standard methods (Association of Official Analytical Chemists [AOAC], 2001). Yttrium (Y) was measured by inductivity couple plasma mass spectroscopy as introduced by Refstie et al. (1997).

### Measurement of Immune Response Parameters

After the feeding trial, shrimp were starved for 24 h. The whole number and total body weight of shrimp in each tank were calculated, weighed, and recorded. Hepatopancreas samples were collected and frozen in liquid nitrogen. Antioxidant and immune response parameters were measured according to the method described by Niu et al. (2018) and Xie et al. (2018).

### DNA Extraction and 16S DNA Gene Sequencing

Total DNA of microbes in intestine was extracted directly with the E.Z.N.A. Stool DNA Kit (OMEGA, United States) according to manufacturer’s instructions. DNA samples were sent to Novogene (Beijing, China) to carry out the 16S rRNA high-throughput sequencing.

### WSSV Injection Challenge Test-Trial 2

The WSSV stemmed from infected *L. vannamei* shrimp imported from Thailand in 1996. The stock solution of virus was produced according to the method described by Niu et al. (2018). Fifteen shrimps from each treatment were challenged for six consecutive days until totally three shrimp were left surviving in any of the treatments to ensure sufficient samples for subsequent analysis. Mortality in each tank was recorded daily. After the WSSV injection challenge test, hepatopancreas and gut samples were collected and frozen in liquid nitrogen for further analysis.

### Analysis for Intestinal Immune-Related Genes Expression

Total RNA extraction, reverse transcription, and quantitative real-time PCR were conducted by the method described by Xie et al. (2018). The primers are shown in Table 3. β-Actin were used as the reference gene. qPCR were performed and quantified on the LightCycler 480 (Roche Applied Science, Basel, Switzerland) according to the manufacturer’s instructions.

**Table 3:** Primer sequences for intestinal cytokines analysis by RT-qPCR.

Primers	Gene name	Primer, forward/reverse (5′–3′)	Accession number or sources
β-Actin	β-Actin	(F) TTTGCGACTCTGGTGATGGT (R) GCGGTGGTGGTGAAAGAATAG	JQ241179
SOD	SOD	(F) GCAATGAATGCCCTTCTACC (R) CAGAGCCTTTCACTCCAACG	Cardona et al., 2015
PO	Prophenoloxidase	(F) GCCTTGGCAACGCTTTCA (R) CGCGCATCAGTTCAGTTTGT	Lai et al., 2005
IκBα	IκBα	(F) CAGCAGACTCCACTCCACTT (R) GAGAGGGGTATTTCCTCGAA	Liu et al., 2017
TGF-β	TGF-β	(F) AACCATGCCCTTGTGCAAAC (R) CTTTGGGGGAACCTCGGTC	Rahimnejad et al., 2018
TNF-α	TNF-α	(F) AAAGAGGAACGTGGTCATGG (R) CACTCCTTTCCCCACTGTGT	Rahimnejad et al., 2018
IL1β	IL1β	(F) GGAGAGGTTAAAGGGTGGCGA (R) TGCCGACTCCAACTCCAACA	NM01123582
IL6	IL6	(F) CCTTGCGGAACCAACAGTTTG (R) CCTCAGCAACCTTCATCTGGTC	HG974247
IL8	IL8	(F) AGAGACACTGAGATCATTGCCAC (R) CCCTCTTCATTTGTTGTTGGC	HG917307

### Calculations and Statistical Analysis

The following variables were calculated:

WG (%) = 100 × (final mean weight - initial mean weight)/initial mean weight.
SGR (% day-1) = 100 × (Ln final mean weight - Ln initial mean weight)/number of days.
Survival rate (%) = 100 × number of final shrimp/number of initial shrimp.
FCR = dry feed intake/wet weight gain.
FCR = dry feed intake/(final body weight - initial body weight).
PER = 100 × (final body weight - initial body weight)/(total amount of the feed × protein content in the feed).
ADCs (%) = 100 × [1 - (trioxide yttrium content in feed/trioxide yttrium content in feces) × (nutrient content in feed/nutrient content in feces*_i_*)].

All data are presented as means ± SEM and subjected to one-way and two-way analysis of variance to test the effects of experimental diets using the software of the SPSS for windows (version 16.0, UAS).

## Results

### Trial 1

#### Growth Performance

As is shown in Table 4, the survival rate was in the scope of 99–100% among all treatments with no significant differences (*P* > 0.05). Growth performance of shrimp fed the D5 diet was significantly higher than that of shrimp fed the control and D4 diets (*P* < 0.05) but without statistically significant difference with other diets (*P* > 0.05). FBW of shrimp in D5 diet was significantly higher than that of other groups, except D3 group (*P* > 0.05). FCR of shrimp fed the control diet was significantly higher than that of shrimp fed D2, D3, and D5 diets (*P* < 0.05) but without significant difference with shrimp fed D4 diet (*P* > 0.05), while PER showed the inverse trend with FCR.

**Table 4:** Effect of five experimental diets on biological performance of *L. vannamei* juveniles.

Items	IBW/g	FBW/g	WG/%	SGR/(%/d)	Survival/%	FCR	PER
D1	0.66 ± 0.01	6.19 ± 0.04^a^	841 ± 8.07^a^	4.00 ± 0.02^a^	100 ± 0.00	1.03 ± 0.01^c^	2.42 ± 0.03^a^
D2	0.65 ± 0.01	6.35 ± 0.06^a^	882 ± 2.56^abc^	4.08 ± 0.01^bc^	99.17 ± 0.83	0.96 ± 0.01^ab^	2.60 ± 0.02^b^
D3	0.66 ± 0.01	6.63 ± 0.06^b^	913 ± 11.94^bc^	4.13 ± 0.02^bc^	100 ± 0.00	0.92 ± 0.02^a^	2.73 ± 0.04^c^
D4	0.64 ± 0.01	6.16 ± 0.09^a^	869 ± 11.70^ab^	4.06 ± 0.02^ab^	99.17 ± 0.83	0.99 ± 0.01^bc^	2.50 ± 0.06^ab^
D5	0.65 ± 0.02	6.58 ± 0.07^b^	921 ± 23.73^c^	4.15 ± 0.04^c^	99.17 ± 0.83	0.92 ± 0.01^a^	2.73 ± 0.02^c^

Values are means ± SEM of three replicates. The superscript small letters (a,b,c) in the same column mean the significant difference at *P* < 0.05.

#### Whole Body Composition

As can be seen in Table 5, the moisture content in control diet was significantly higher than other diets (*P* < 0.05). D5 diet had the highest protein content and was significantly higher than that of shrimp fed other diets (*P* < 0.05). The lipid content of shrimp in D4 was significantly higher than that in D1, D3, and D5 (*P* < 0.05) but without significant difference with shrimp fed D2 diet (*P* > 0.05). The ash content of shrimp fed the control diet was significantly lower than that of shrimp fed the other four macro-algaes diets (*P* < 0.05); however, no significant difference was found in ash content among the four macro-algaes diets treatments (*P* > 0.05).

**Table 5:** Effect of five experimental diets on whole body composition (%) of *L. vannamei* juveniles.

Items	D1	D2	D3	D4	D5
Whole body					
Moisture	75.91 ± 0.03^d^	75.25 ± 0.03^b^	75.15 ± 0.03^a^	75.39 ± 0.04^c^	75.28 ± 0.01^b^
Protein	72.46 ± 0.03^a^	73.60 ± 0.01^c^	73.69 ± 0.04^c^	73.29 ± 0.04^b^	73.80 ± 0.04^d^
Lipid	5.92 ± 0.04^a^	6.59 ± 0.04^cd^	6.45 ± 0.07^bc^	6.66 ± 0.10^d^	6.38 ± 0.03^b^
Ash	13.81 ± 0.03^a^	14.30 ± 0.15^b^	14.32 ± 0.03^b^	14.30 ± 0.11^b^	14.34 ± 0.05^b^

*Values are means ± SEM of three replicates based on dry matter. The superscript small letters (a,b,c,d) in the same row mean the significant difference at *P* < 0.05.*

#### Apparent Digestibility Coefficiency

Apparent digestibility coefficiencts of dry matter from the D2, D3, and D5 treatments were higher than that from the control and D4 treatments (*P* < 0.05) (Table 6). ADC of protein from the D5 treatment showed the highest value and was significantly higher than that from the control, D2, and D4 treatment (*P* < 0.05) while with no significant difference with the D3 treatment (*P* > 0.05). ADC of lipid from the D4 treatment was significantly lower than that from the other treatments (*P* < 0.05), but there is no statistical significant difference with the other four treatments (*P* > 0.05).

**Table 6:** ADCs (%) of *L. vannamei* juveniles in five experimental diets.

Items	D1	D2	D3	D4	D5
Digestibility					
Dry matter	78.67 ± 0.33^a^	85.67 ± 0.67^c^	85.33 ± 0.67^c^	82.33 ± 0.33^b^	85.67 ± 0.33^c^
Protein	79.67 ± 0.88^a^	83.67 ± 0.33^b^	84.67 ± 0.33^bc^	80.33 ± 0.33^a^	85.67 ± 0.33^c^
Lipid	97.33 ± 0.33^b^	97.67 ± 0.33^b^	97.33 ± 0.88^b^	91.67 ± 0.88^a^	97.67 ± 0.33^b^

*Values are means ± SEM of three replicates. The superscript small letters (a,b,c) in the same row mean the significant difference at *P* < 0.05.*

#### Immune Response

There was no significant differences in SOD, MDA, and carbonyl protein contents activity among all diets treatments (*P* > 0.05) in trail 1 (Table 7). While TAS and ProPO activities of shrimp fed the macroalgae-containing diets were significantly higher than those of shrimp fed the control diet (*P* < 0.05), and no significant differences were found in TAS and PO activities among shrimp fed the four macroalgae-containing diets (*P* > 0.05).

**Table 7:** Effect of experimental diets on immune response (TAS, μmol g^-1^ organ; SOD, U mg^-1^ protein; PO, O.D.490 nm; MDA, nmol mg^-1^ protein; carbonyl protein, nmol mg^-1^ protein) of *L. vannamei* both during the 8 weeks rearing period and after the WSSV injection challenge test.

Diets	Trial1: During the 60-day rearing period					Trial 2: After the challenge test				
	D1	D2	D3	D4	D5	D1	D2	D3	D4	D5
Hepatopancreas antioxidant parameters										
TAS	2.34 ± 0.04 ^aB^	4.20 ± 0.03 ^bB^	4.22 ± 0.05 ^bB^	4.21 ± 0.02 ^bB^	4.20 ± 0.05 ^bB^	1.97 ± 0.04 ^aA^	3.18 ± 0.02 ^cA^	3.19 ± 0.01 ^cA^	2.55 ± 0.05 ^bA^	3.21 ± 0.03 ^cA^
SOD	2.27 ± 0.04 ^A^	2.29 ± 0.09 ^A^	2.32 ± 0.04 ^A^	2.29 ± 0.07 A	2.30 ± 0.02 A	4.22 ± 0.03 cB	3.14 ± 0.02 aB	3.15 ± 0.04 aB	3.34 ± 0.06 ^bB^	3.14 ± 0.02 ^aB^
PO	5.16 ± 0.04 ^aA^	6.78 ± 0.06 ^bA^	6.82 ± 0.07 ^bA^	6.79 ± 0.05 ^bA^	6.80 ± 0.05 ^bA^	7.38 ± 0.07 ^aB^	10.26 ± 0.04 ^cB^	10.24 ± 0.04 ^cB^	9.61 ± 0.08 ^bB^	10.21 ± 0.18 ^cB^
Hepatopancreas oxidative injury parameters										
MDA	12.33 ± 0.09 A	12.27 ± 0.10 A	12.26 ± 0.07 A	12.26 ± 0.05 A	12.26 ± 0.04 A	32.26 ± 1.08 ^bB^	21.54 ± 0.47 aB	21.89 ± 0.23 aB	21.97 ± 0.44 aB	22.55 ± 0.23 aB
Carbonyl	1.22 ± 0.02 A	1.19 ± 0.05 A	1.21 ± 0.04 A	1.22 ± 0.03 A	1.19 ± 0.04 A	2.90 ± 0.04 cB	1.70 ± 0.03 aB	1.71 ± 0.04 aB	2.19 ± 0.02 ^bB^	1.69 ± 0.03 aB
protein

*Values are means ± SEM of three replicates. The superscript small letters (a,b,c) in the same row mean the significant difference at *P* < 0.05 within the same trial. The superscript capital letters (A,B) in the same row mean the significant difference at *P* < 0.05 of the same dietary treatment between trial 1 and trial 2 (such as comparison of P0 between trial 1 and trial 2).*

#### Intestinal Immune-Related Genes Expression

**FIGURE 1:**
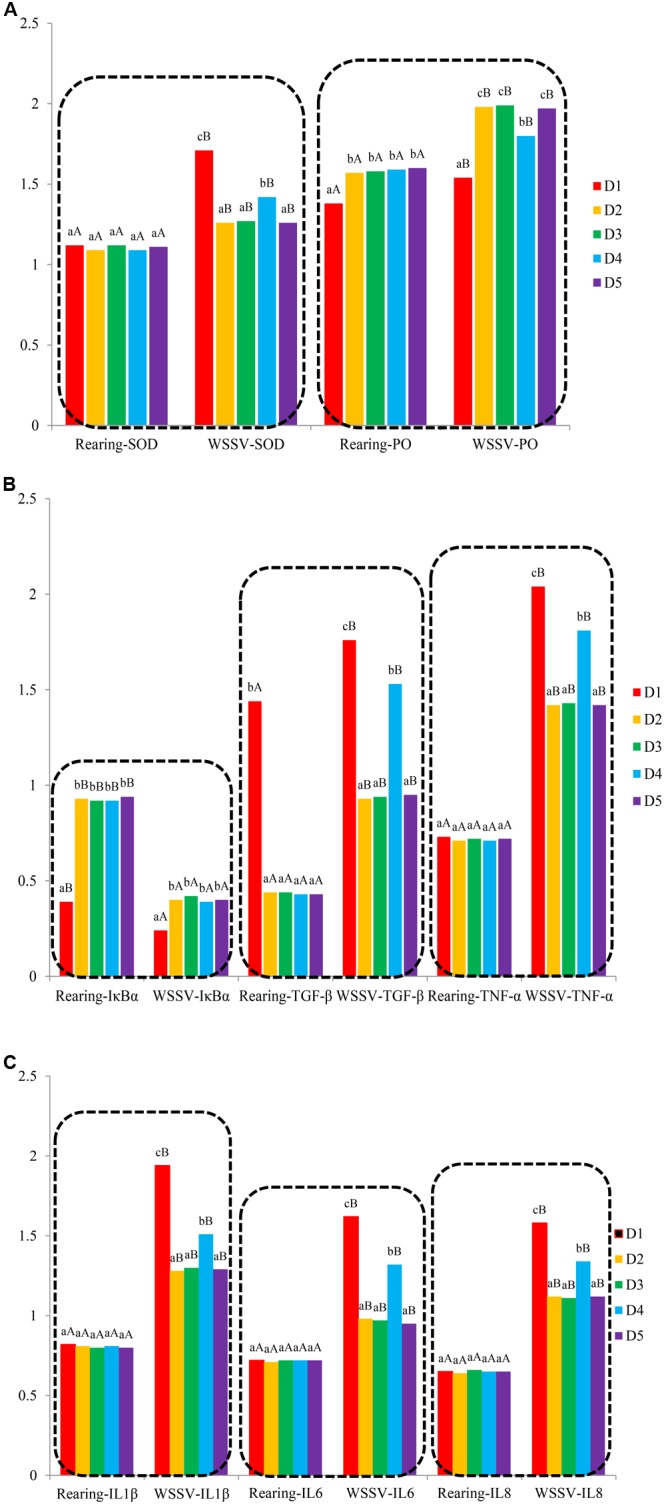
Analysis of intestinal immune-related genes expression [**A**: SOD and PO genes expression levels; **B**: IκBα, TGF-β, and TNF-α genes expression levels; **C**: IL1β, IL6, and IL8 genes expression levels. Different letters indicated significant differences between groups (*P* < 0.05)].

In trail 1, ProPO, IκBα, and TGF-β genes expression levels of shrimp fed the macroalgae-containing diets were significantly higher than those of shrimp fed the control diet (*P* < 0.05), while no significant differences were found in SOD, TNF-α, IL1β, IL6, and IL8 genes expression levels among all diets treatments (*P* > 0.05) (Figure 1).

#### Intestinal Microbiota Structures of *L. vannamei*

As shown in Table 8, the numbers of OTUs in the macroalgae-containing groups were increased compared with the control group (*P* < 0.05) and the highest number of OTUs was found in the D5 group. The tendency of Shannon index was the same with OTUs. Shannon index of D5 group was the highest and D1 group was the lowest among all the groups. With regard to the Chao 1 index, the highest Chao 1 index was found in D5 group, then followed by the D2 and D3 groups and finally the D1 and D4 groups.

**Table 8:** Diversity index of gut bacteria of *L. vannamei* juveniles fed the five experimental diets for 8 weeks based on V4 sequences.

Items	D1	D2	D3	D4	D5
OTUs	283 ± 3.06^a^	360 ± 2.00^c^	390 ± 3.46^d^	311 ± 1.73^b^	522 ± 2.31^e^
Chao 1	232 ± 11.82^a^	323 ± 19.34^b^	302 ± 17.87^b^	252 ± 11.75^a^	551 ± 20.85^c^
Shannon	4.45 ± 0.01^a^	4.94 ± 0.01^c^	5.06 ± 0.01^d^	4.81 ± 0.01^b^	5.32 ± 0.02^e^

*Values are means ± SEM (*n* = 3 for OTUs and *n* = 10 for Chao1 and Shannon). Means in the same row without a common superscript letter differ, *P* < 0.05 (one-factor ANOVA, Duncan’s multiple range test). OTU, operational taxonomic unit; Chao1, Chao 1 index; Shannon, Shannon diversity index.*

**FIGURE 2:**
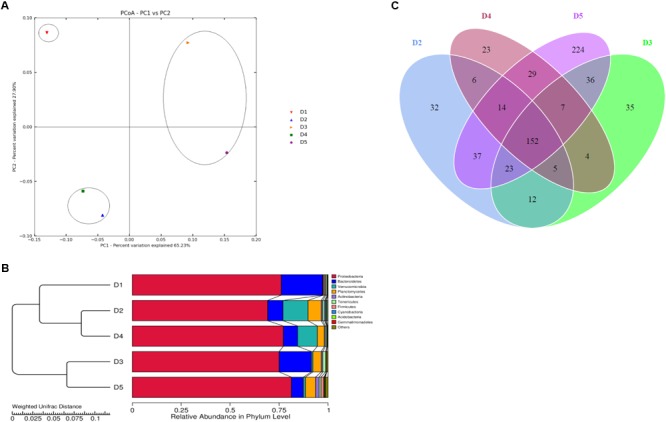
Analysis of intestinal microbiota structure of *L. vannamei* juveniles from different diets treatments (**A**: PCoA; **B**: UPGMA clustering tree; **C**: OTU-Venn).

**FIGURE 3:**
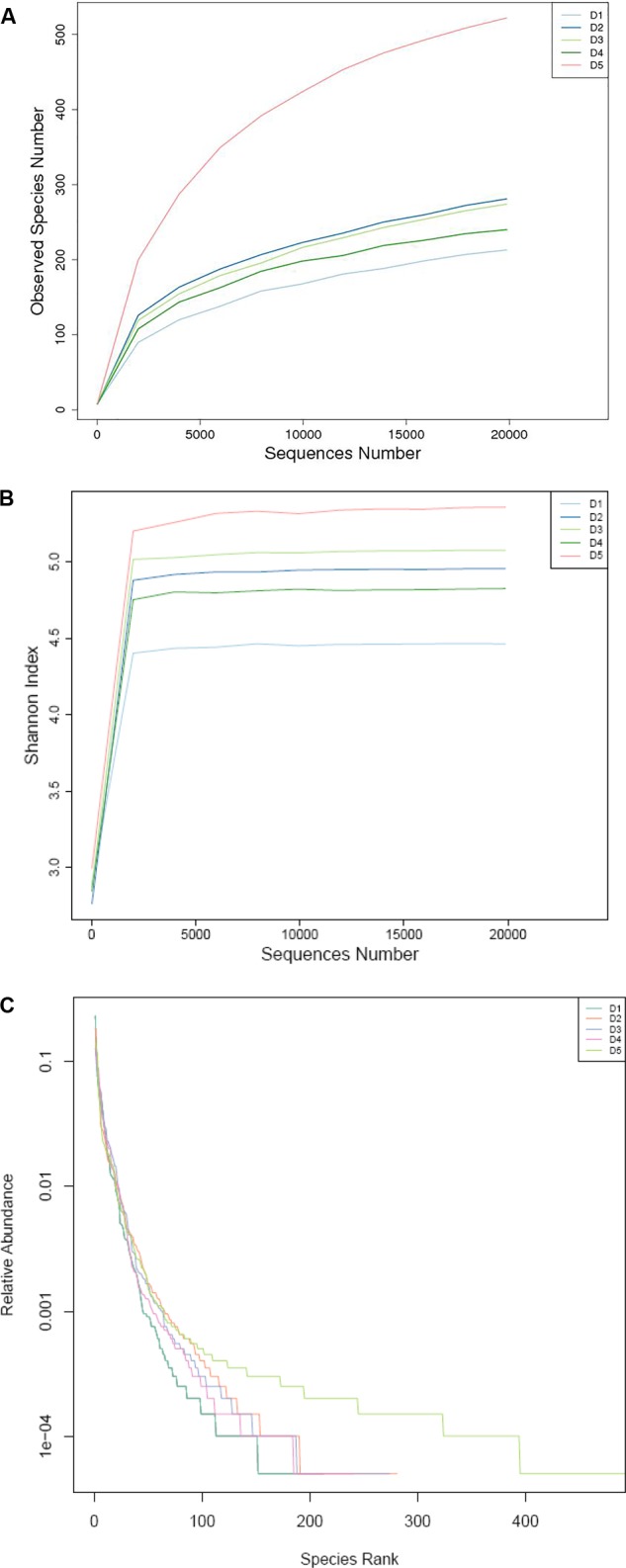
**(A)** Observed species number, **(B)** Shannon index, and **(C)** relative abundance based on the bacterial community 16S rDNA sequences.

Intestinal microbiota in *L. vannamei* juveniles was clearly divided into three clusters based on the analysis of PCoA (Figure 2A) and UPGMA clustering (Figure 2B). The principal coordinates 1 and 2, respectively, explained 70.52 and 30.16% of the total structure variations. The clustering analysis showed that D3 and D4 groups were clustered together and D2 and D5 groups were clustered together and separated from the control group. The distributions in the quantities of OTUs among the groups were presented in OTU-Venn (Figure 2C). In addition, the rarefaction curves (Figures 3A–C) all reached a plateau, suggesting that the sequencing depth for all samples was well to cover intestinal bacterial community diversity.

#### Changes in the Compositions of Intestinal Microbiota

**FIGURE 4:**
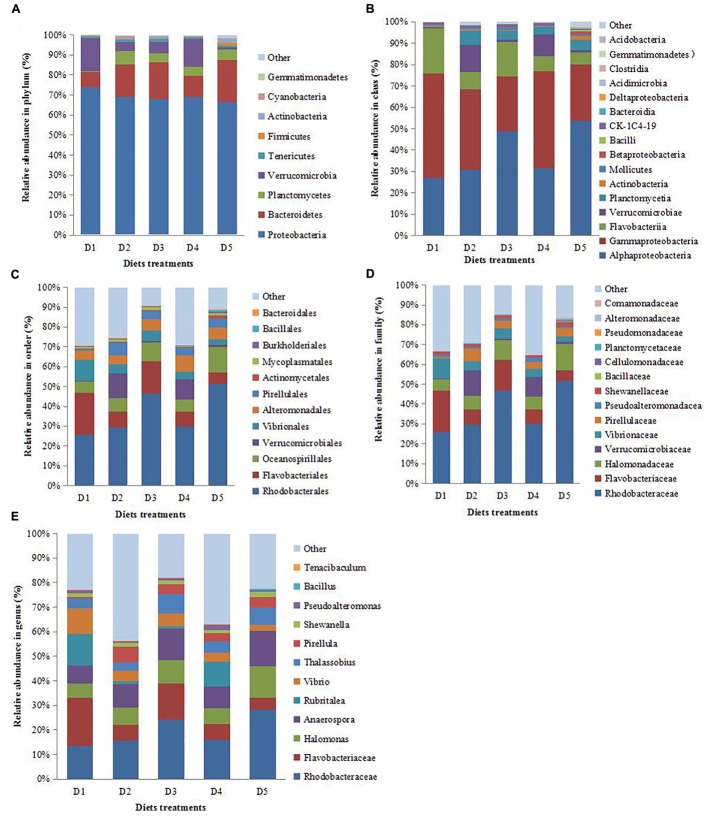
Composition and relative abundance of bacterial communities based 16S rDNA sequences. Pattern **A–E** indicates the composition and relative abundance of bacterial communities in phylum, class, order, family, and genus level, respectively.

As Figure 4A showed that *Proteobacteria* was the most abundant in control group followed by *Bacteroidetes, Tenericutes, Planctomycetes*, and *Verrucomicrobia*. D2, D3, and D5 groups evidently increased the proportion of *Bacteroidetes* and decreased the proportion of *Proteobacteria* and *Verrucomicrobia* compared with the D1 and D4 groups.

Figure 4B showed that the proportion of *Alphaproteobacteria* was obviously increased while *Gammaproteobacteria* was decreased in D3 and D5 groups when compared with those in D1, D2 and D4 groups. The addition of dietary macro-algaes increased the proportion of *Planctomycetia*. D2 and D4 groups evidently increased the proportion of *Verrucomicrobiae* compared with other groups.

Figure 4C showed that the proportions of *Rhodobacterales* and *Oceanospirillales* were obviously increased in D3 and D5 groups when compared with those in D1, D2 and D4 groups. The addition of dietary macro-algaes decreased the proportion of Vibrionales while increased the proportion of *Pirellulales*. D2 and D4 groups evidently increased the proportion of *Verrucomicrobiales* compared with other groups.

Figure 4D showed that dietary macro-algaes obviously increased the proportion of *Rhodobacteraceae, Halomonadaceae*, and *Pirellulaceae* while decreased the proportion of *Flavobacteriaceae* and *Vibrionaceae* compared with those in the control group. Moreover, the lowest proportion of *Flavobacteriaceae* and *Vibrionaceae* was found in D5 group and *Bacillaceae* and *Alteromonadaceae* were found only in the D5 group.

Figure 4E illustrated that *Flavobacteriaceae* was the most abundant bacteria in D1 group followed by *Rhodobacteraceae, Rubritalea*, and *Vibrio*. Rhodobacteraceae became the predominant genus in D2 and D4 groups followed by *Anaerospora, Rubritalea, Halomonas*, and *Flavobacteriaceae*, while the relative abundance of *Rubritalea* in the D4 group was obviously higher than that in the D2 group. *Rhodobacteraceae* was also the predominant genus in D3 and D5 groups followed by *Flavobacteriaceae, Halmonas, Anaerospora, Thalassobius, Vibrio*, and *Priellula*, while relative abundance of *Flavobacteriaceae* and *Vibrio* in the D5 group was obviously lower than those in the D3 group. In addition, *Bacillus* was found only in the D5 group. As mentioned above, macro-algaes actively modified the intestinal microbiota, including composition and community structure.

**FIGURE 5:**
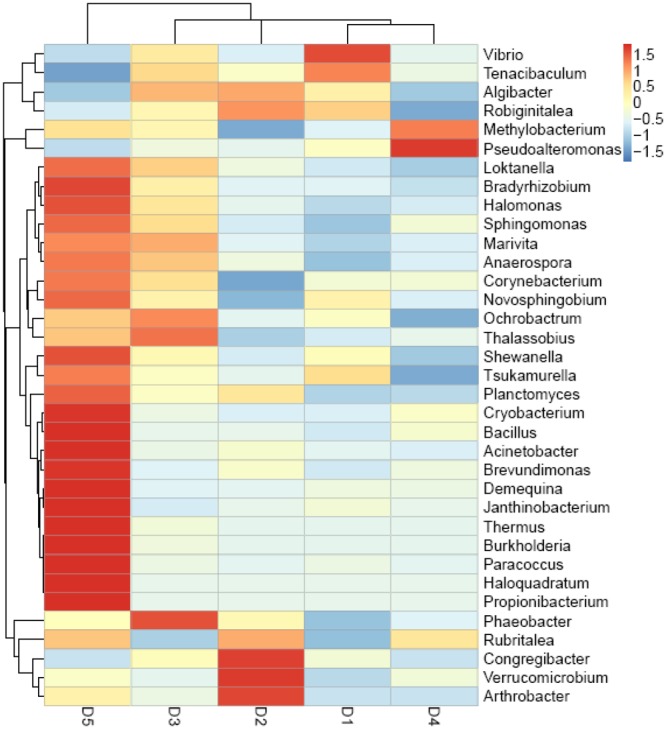
Heatmap analysis of the species abundance clustering in the top 35 genus level based on the bacterial community 16s rDNA sequence.

Figure 5 showed that the top 35 genera from D5 diet differed from other diets, especially D1, D3, and D4 treatments. Besides, *Vibrio* in D1 diet showed the highest content compared to the macro-algaes supplemented diets. The PCoA (Figure 2A) illustrated microbiota in shrimp fed D2 and D5 diets were similar, and the macro-algaes supplemented diets were different from the control diet in microbial community composition.

### Trial 2

#### Mortality of Shrimp During WSSV Injection Challenge Test

**FIGURE 6:**
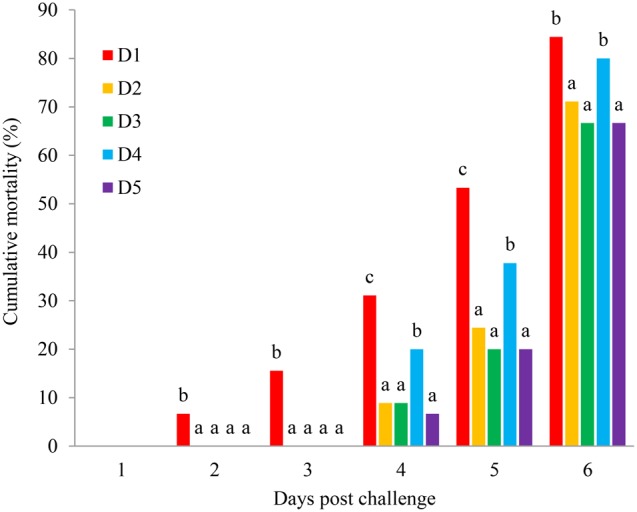
Mean ± SD (*n* = 3) values of cumulative mortality (%) of *L. vannamei* after challenging with WSSV. D1, control diet; D2, *Porphyra haitanensis* diet; D3, *Undaria pinnatifida* diet; D4, *Saccharina japonica* diet; D5, *Gracilaria lemaneiformis* diet.

As can be seen from Figure 6, there was no shrimp dead in all treatments in the first day. Shrimp started to die from the second day, and the mortality of shrimp in D1 was significantly higher than that of shrimp fed other diets during 2–3 challenged days (*P* < 0.05). Besides, lots of died in the fourth day and the mortality of shrimp in D2, D3, and D5 groups were significantly lower than that in D1 and D4 groups during 4–6 challenged days (*P* < 0.05). The death rate was found lower in shrimp fed the D2, D3, and D5 groups after the challenge test.

#### Immune Response

The SOD activity of shrimp in D2, D3, and D5 groups was significantly lower than that of shrimp in D1 and D4 groups (*P* < 0.05) (Table 7). TAS and ProPO activities showed the reverse trend with SOD activity. TAS and ProPO activities of shrimp fed the D2, D3, and D5 groups were significantly higher than those of shrimp fed the D1 and D4 groups (*P* < 0.05). The MDA contents of shrimp control diet was significantly higher than that of shrimp fed other four macroalgaes-containing diets (*P* < 0.05), while carbonyl protein content in D1 and D4 groups was significantly higher than that in D2, D3, and D5 groups (*P* < 0.05).

#### Intestinal Immune-Related Genes Expression

Intestinal immune-related genes expressions are shown in Figure 1. In trial 2, ProPO gene expression level in D2, D3, and D5 groups was significantly higher than those in D1 and D4 groups (*P* < 0.05), and the lowest value was found in D1 group. SOD, IκBα, TGF-β, TNF-α, IL1β, IL6, and IL8 genes expression levels showed the same tendency. SOD, IκBα, TGF-β, TNF-α, IL1β, IL6, and IL8 genes expression levels in D2, D3, and D5 groups were significantly lower than those in D1 and D4 groups (*P* < 0.05), and the highest values of these genes expression levels were found in D1 groups.

### Comparison of Immune Response and Immune-Related Genes Expression Between the Two Trials

Total antioxidant status of shrimp in trial 2 showed lower activities compared with shrimp in trial 1, while SOD and PO activities as well as MDA and carbonyl protein contents exhibited the opposite tendency (Table 7).

As for the comparison of immune-related genes expression (Figure 1) between the two trials, PO, SOD, IκBα, TGF-β, TNF-α, IL1β, IL6, and IL8 genes expression levels of shrimp from trial 1 were lower compared to the shrimp from trial 2 (*P* < 0.05).

## Discussion

### Effect of the Four Species of Macro-Algaes on Growth Performance

A variety of macro-algaes, including *Palmaria mollis* (Demetropoulos and Langdon, 2004), *Gracilaria bursa-pastoris, Ulva rigida* and *Gracilaria cornea* (Valente et al., 2006), *Gracilaria fisheri* (Kanjana et al., 2011), UP (Niu et al., 2015), GL (Yu et al., 2016), and PH (Niu et al., 2018) have been considered as promising choices because of the availability of nutrients for aquatic feeds. The present results showed that shrimp fed the GL diet had the highest growth performance and were significantly higher than shrimp fed the control and the SJ diets, which suggested that dietary GL is more suitable as a feed ingredient than the other three macro-algaes. In the study of Valente et al. (2006), the growth promoting effect of *G. bursa-pastoris* in European sea bass (*D. labrax*) juveniles was superior to *G. cornea* and *U. rigida*. The results suggested that there is a species-specific response for aquatic animals to algae and indicated that low level inclusion of macro-algaes in diets exerted a general beneficial effect on shrimp. The relative low nutritive value of macro-algae such as the present SJ could be due to the low dietary protein or carotenoids level or high ash content.

### Effect of the Four Species of Macro-Algaes on Apparent Digestibility Coefficients

Apparent digestibility coefficients of dry matter supply measurement of overall quantity of the digested and absorbed ingredient (Brunson et al., 2015). The results showed that the overall digestibility was influenced by various kinds of marco-algaes. In the study of Xu et al. (2011), rabbitfish *Siganus canaliculatus* fed diets containing 33% dried GL showed no significant differences in the ADC of dry matter and protein with fish fed control diet. This suggests that dietary GL is more suitable as an ingredient in aquatic animals.

In the present study, shrimp fed the PH, UP, and GL diets had the higher ADC of dry matter compared to shrimp fed the SJ diet. The relatively lower ADC of dry matter of SJ could be explained by the more indigestible polysaccharides and ash compounds in the ingredient which are difficulty digested by *L. vannamei* as shown in other studies (Sullivan and Reigh, 1995; Stone et al., 2000; Lee, 2002; Zhou et al., 2004).

It seems clear that high polysaccharide (Burtin, 2003), fiber (Leary and Lovell, 1975), and carbohydrate (Appler, 1985) contents in macro-algaes influence the digestibility. On the contrary, the higher ADC of dry matter of PH, UP, and GL could attribute to the more digestible starch compounds that can be absorbed better by shrimp. SJ meal had the highest ash content, fiber structures may act as the physical hindrances between digestive enzymes in the digestive tract and nutrients, leading to lesser availability (Potty, 1996; Appler, 2010).

The present experiment showed that the highest ADC of protein was in shrimp fed the GL and UP diets and the lowest ADC of protein in shrimp fed the control and SJ diets. The high ADC of protein is probably because of the well-balanced amino acid profile in GL and UP diets (Andrews and Page, 1974).

### Effect of the Four Species of Macro-Algaes on Immune Response

Aspartate aminotransferase (AST) activity in shrimp hepatopancreas suggested the health status; moreover, SOD is used to scavenge kinds of reactive oxygen, preventing tissues from radical damage (Pan et al., 2003). The ProPO activity can contribute to the activation of the melanin synthesis pathway, resulting in pathogens to be killed (Palmer et al., 2011), which means that shrimp may be more resistant to infection with higher ProPO activity (Newton et al., 2004). When shrimp were challenged with WSSV, TAS activity significantly decreased while SOD, ProPO activities, and MDA content increased compared with the normal rearing trial. The results suggested that WSSV induced the oxidative damage to hepatopancreas, and hepatic antioxidant defense was partly destroyed. It has been proved that PH can enhance the immunity by immune enzyme activity (Niu et al., 2018). The previous study suggested brown seaweed *Sargassum wightii* has the antioxidant roles to pancreatitis induced by oxidative stress (Immanuel et al., 2012). Dietary macro-algaes in the present study significantly increased TAS and ProPO activities and reduced MDA levels, as well as promoted the expression level of ProPO in shrimp. These results indicated that the optimal inclusion of macro-algaes would contribute to attenuate hepatic oxidative damage through strengthening hepatic antioxidant capacity.

### Effect of the Four Species of Macro-Algaes on Intestinal Immune-Related Genes Expression

As an immunological organ, intestine tolerates to commensal antigens and responds to pathogenic stimuli (Brown et al., 2012). This study provides evidence that macro-algaes regulated the expression of immune-related genes effectively, such as IκBα, TGF-β, TNF-α, IL1β, IL6, and IL8. After the WSSV injection challenge test, IκBα was upregulated in shrimps fed diets supplemented with macro-algaes compared with those fed with the control; however, the expressions of TGF-β, TNF-α, IL1β, IL6, and IL8 were downregulated in the intestine (Figure 1).

In NF-κB signal pathway, an inactive heterodimer was inhibited by kappa B (IκB) to sequestered in the cytoplasm. Once activated, the IκB was degraded and the active heterodimer translocated into the nucleus and activated the target genes.

The results showed that after WSSV injected, TNF-α, IL1β, IL6 and IL8 were upregulated, suggesting that NF-κB signal pathway was activated by degenerated IκBα (Kavitha et al., 2013). The IκBα was higher in shrimps fed with macro-algaes supplemented diets compared with those fed with the control meaning that macro-algaes were anti-inflammatory, which was proved by the expression of the inflammatory factor.

Recent research suggested that NF-κB was activated by ER stress during the inflammation (Kitamura, 2011). Normally, NF-κB is inactively bound by its inhibitor, IκBα. Once the IκBα kinase (IKK) phosphorylates IκBα, resulting in its degradation, the NF-κB will be activated (Ko et al., 2017). In this study, the up-regulated IκBα expression was shown in shrimp fed macro-algaes supplemented diets. These findings indicated that dietary macro-algaes enhanced intestinal immunity of shrimp, and immune-enhancement of macro-algaes could partly be associated with the inhibition of NF-κB activation in intestine. Further, NF-κB activation could promote more transcription of inflammatory cytokines mainly including TNF-α and IL1β. TNF-α and IL1β are two proinflammatory cytokines, which were used to indicate whether inflammatory response occurred or not (Secombes et al., 2001). The presence of these inflammatory cytokines in turn amplify NF-κB signaling, resulting in the feedback loops to aggravate tissue damage (Ren et al., 2016). Several studies have been proved that exogenous stimulus such as lipopolysaccharide caused the inflammation response in intestine, represented by the increments in expression level of TNF-α, IL1β, IL6, and IL8 (Djordjevic et al., 2009; Pérez-Sánchez et al., 2015; Yashaswini et al., 2017). Similarly, WSSV injection led to the high expression of IL1β, IL6, IL8, and TNF-α in this study.

Normally, TGF-β functioned bifunctionally which rely on the context, manifesting the roles of inducing and aggravating local tissue inflammation response or initiating anti-inflammation response (Chung et al., 2010). In this study, WSSV challenge stimulated the increment in expression level of TGF-β, drastically aggravating inflammation response in intestine. While during the normal rearing period, the increases in TGF-β expression levels in macro-algaes groups demonstrated the certain anti-inflammation activity of macro-algaes. Similar results were found in common carp (Wang et al., 2015) and *Labeo rohita* (Giri et al., 2015) fed with immunostimulants.

### Effect of the Four Species of Macro-Algaes on Intestinal Microbiota

Intestinal microbiota supplies the host nutritional and energy, acts as a pathogenic barrier, and exerts great influence on the maintenance of immune homeostasis (Sekirov et al., 2010). Microbial balance in intestine is helpful to decrease NF-κB activation and retain the epithelial barrier function (Sachdev and Pimentel, 2013). Dietary macro-algaes increased α-diversity of microbes in intestine compared with the control group in this study. In addition, the highest Shannon index was observed in GL diet group, and the Shannon index ranking from big value to small value is GL, UP, PH, SJ, and control diet group, which might be associated with the different macro-algae sources. Other studies have been proved that diet and stress response very easily altered intestinal microbiota community composition, disturb intestinal homeostasis, and influence anti-inflammation response (Sachdev and Pimentel, 2013). The present results suggested that some unknown immunostimulants contained in the macro-algaes could act as the prebiotic-like role to decrease NF-κB activation and retain microbial homeostasis in intestine. Immunostimulants could hinder the invasion of pathogens by ways of enhancing microbial diversity and ease microbial disturbance (Seifried et al., 2007).

Intestinal microbiota homeostasis of shrimp was maintained by dietary macro-algaes addition, evidently represented by the decreasing *Proteobacteria* and increasing *Bacteroidetes* in this study. However, *Gammaproteobacteria* from PH, UP, and GL diets groups were lower than that from the SJ and control diets groups. Commonly, *Proteobacteria* and *Gammaproteobacteria* involved in intestinal pathogenesis and resulted in intestinal dysbiosis (Schippa and Conte, 2014). The present study showed that macro-algaes could evidently decrease detrimental bacteria within *Gammaproteobacteria*. Dietary macro-algaes obviously reduced the growth of *Flavobacteria, Proteobacteria*, and *Gammaproteobacteria* and increased the proliferation of *Bacteroidetes* and *Firmicutes* especially from the GL diet group. Besides, *Bacillaceae* was increased while *Vibrionaceae* was decreased after dietary macro-algaes. These results indicated that macro-algae especially GL regulated intestinal community composition and improved intestinal homeostasis. It was reported that *Bacteroidetes* could be associated with NF-κB activation, and its proliferation is conducive to declining inflammatory cytokine levels (Stringer, 2013). Moreover, *Clostridia* was only found in GL diet group.

Clostridia is good for the development of intestinal epithelial cells and energy metabolism. Besides, Clostridia plays a role in fermenting carbohydrate into conjugated linoleic acid and/or butyrate, which is beneficial for intestinal epithelial cells proliferation and differentiation (Chen et al., 2011). Importantly, butyrate is vital for the generation of specific T lymphocytes, further strengthening the suppression of inflammation response and lowering pathogen-induced barrier–function disruption (Schippa and Conte, 2014). Therefore, intestinal microbes and their products played the crucial roles in the modulation of intestinal immune response. Intestinal microbiota is closely associated with the development of immune system. As the above that suitable macro-algae modified intestinal microbiota as prebiotics.

In this study, intestinal microbiota community compositions of shrimp fed different macro-algae were significantly different, as well as the regulation of intestinal immune responses. Macro-algaes have attracted the increasing attention in aquatic animals owing to its unknown immunostimulants and eco-friendly characteristics (Michel and Macfarlane, 1996). Kaushik et al. (2008) revealed that *Nostoc commune* can yield antibacterial compounds antagonized a series of bacterial species. The present results including the WSSV injection challenge test suggested that dietary GL, PH, or UP might make a difference on shrimp health by inhibiting the pathogenic bacteria, improving nutritional contribution, and enhancing immune responses. It is well known that various factors such as concentrations of macro-algae supplemented in the diet, species-specific response, and duration of feeding may affect the immune response of aquatic animals (Valente et al., 2006; Yu et al., 2016). Moreover, the mechanism that dietary macro-algaes regulated the intestinal microbiota composition and further maintained the intestinal homeostasis needs to be studied further.

## Conclusion

Dietary macro-algaes especially GL, PH, or UP attenuated the oxidative damage to intestine in *L. vannamei* through elevating TAS, PO activities, decreasing SOD activity, MDA, and carbonyl protein contents, and correspondingly up-regulating PO and down-regulating SOD expression levels. Dietary macro-algaes supplementation improved intestinal immunity by up-regulating the expression of IκBα and down-regulating those of TGF-β, TNF-α, IL1β, IL6, and IL8. In addition, dietary macro-algaes modified intestinal microbiota of *L. vannamei*, symbolized by enhancing the relative abundance of the proportion of *Bacteroidetes, Firmicutes*, and *Bacillaceae*, whereas decreasing those of *Gammaproteobacteria* and *Vibrionaceae*. The results suggested that dietary suitable macro-algaes could be used as one kind of functional ingredients or acted as prebiotic-like role for preventing intestinal oxidative damage in shrimp whether under the normal rearing or the WSSV challenge conditions.

## Ethics Statement

All experimental procedures were conducted in conformity with institutional guidelines for the care and use of laboratory animals in Sun Yat-sen University, Guangzhou, China, and conformed to the National Institutes of Health Guide for Care and Use of Laboratory Animals (Publication No. 85-23, revised 1985).

## Author Contributions

JN, Y-JL, and L-XT designed the study. J-JX, T-YG, and S-WX carried out the rearing work. J-JX, H-HF, S-YL, Y-MZ, and JN analyzed the results and JN and J-JX wrote the manuscript with contributions from the other authors.

## Conflict of Interest Statement

The authors declare that the research was conducted in the absence of any commercial or financial relationships that could be construed as a potential conflict of interest.

## Abbreviations

ADC: apparent digestibility coefficient
FBW: final body wet weight
FCR: feed conversion ratio
IBW: initial body wet weight
IκBα: nuclear transcription factor inhibitor
IL1β: interleukin 1β
IL6: interleukin 6
IL8: interleukin 8
PCoA: principal coordinates analysis
MDA: malondialdehyde
PER: protein efficiency ratio
PO: phenoloxidase
SOD: superoxide dismutase
SGR: specific growth rate
TGF-β: transforming growth factor β
TNF-α: tumor necrosis factor α
WG: weight gain
